# Indications and Strategy for Active Surveillance of Adult Low-Risk Papillary Thyroid Microcarcinoma: Consensus Statements from the Japan Association of Endocrine Surgery Task Force on Management for Papillary Thyroid Microcarcinoma

**DOI:** 10.1089/thy.2020.0330

**Published:** 2021-02-12

**Authors:** Iwao Sugitani, Yasuhiro Ito, Dai Takeuchi, Hirotaka Nakayama, Chie Masaki, Hisakazu Shindo, Masanori Teshima, Kazuhiko Horiguchi, Yusaku Yoshida, Toshiharu Kanai, Mitsuyoshi Hirokawa, Kiyomi Y. Hames, Isao Tabei, Akira Miyauchi

**Affiliations:** ^1^ Department of Endocrine Surgery, Nippon Medical School Graduate School of Medicine, Tokyo, Japan.; ^2^ Department of Surgery, Kuma Hospital, Kobe, Japan.; ^3^ Department of Breast and Endocrine Surgery, Nagoya University, Nagoya, Japan.; ^4^ Department of Surgery, Yokohama City University, Yokohama, Japan.; ^5^ Department of Surgery, Ito Hospital, Tokyo, Japan.; ^6^ Department of Surgery, Yamashita Thyroid Hospital, Fukuoka, Japan.; ^7^ Department of Otolaryngology, Head and Neck Surgery, Kobe University, Kobe, Japan.; ^8^ Division of Endocrinology and Metabolism, Department of Internal Medicine, Gunma University, Maebashi, Japan.; ^9^ Department of Breast and Endocrine Surgery, Tokyo Women's Medical University, Tokyo, Japan.; ^10^ Division of Breast and Endocrine Surgery, Department of Surgery, Shinshu University School of Medicine, Matsumoto, Japan.; ^11^ Department of Pathology, Kuma Hospital, Kobe, Japan.; ^12^ Department of Surgery, The Jikei University School of Medicine, Tokyo, Japan.

**Keywords:** papillary thyroid microcarcinoma, active surveillance, indications, strategy, task force consensus statements, Japan Association of Endocrine Surgery

## Abstract

***Background:*** The question of how to manage patients with low-risk papillary thyroid microcarcinoma (PTMC; T1aN0M0) has recently become an important clinical issue. Two Japanese centers have conducted prospective clinical trials of active surveillance (AS) for low-risk PTMC since the 1990s, reporting favorable outcomes. This policy has thus seen gradual adoption worldwide to avoid overtreatment. Not all PTMCs are suitable for AS, however, and many physicians still hesitate to apply the management policy in daily clinical practice. A task force on management for PTMC created by the Japan Association of Endocrine Surgery collected and analyzed bibliographic evidence and has produced the present consensus statements regarding indications and concrete strategies for AS to facilitate the management of adult patients diagnosed with low-risk PTMC.

***Summary:*** These statements provide indications for AS in adult patients with T1aN0M0 low-risk PTMC. PTMCs with clinical lymph node metastasis, distant metastasis, recurrent laryngeal nerve (RLN) paralysis due to carcinoma invasion, or protrusion into the tracheal lumen warrant immediate surgery. Tumors suspected of aggressive subtypes on cytology are recommended for immediate surgery. Immediate surgery is also recommended for tumors adherent to the trachea or located along the course of the RLN. Practical strategies include diagnosis, decision-making, follow-up, and monitoring related to the implementation of AS. The rate of low-risk PTMC progression is lower in older patients. However, we recommend continuing AS as long as circumstances permit. Future tasks in optimizing management for low-risk PTMC are also described, including molecular markers and patient-reported outcomes.

***Conclusions:*** An appropriate multidisciplinary team is necessary to accurately evaluate primary tumors and lymph nodes at the beginning of and during AS, and to adequately reach a shared-decision with individual patients. If appropriately applied, AS of low-risk PTMC is a safe management strategy offering favorable outcomes and preserves quality of life at low cost.

## Introduction

The incidence of thyroid cancer has been rapidly increasing throughout the world (1–3). This increase is generally attributed to the increased detection of small papillary thyroid cancers (PTCs) following the widespread adoption of imaging modalities, such as ultrasonography (US) and US-guided fine-needle aspiration cytology (FNAC). Small PTCs were previously detected mostly only on autopsy. Mortality due to thyroid cancer has remained stable. Thus, there is growing concern for overdiagnosis and overtreatment of small PTCs (1–5).

Two Japanese institutions, Kuma Hospital and the Cancer Institute Hospital (CIH), have conducted prospective clinical studies of active surveillance (AS) for low-risk (T1aN0M0) papillary thyroid microcarcinoma (PTMC) since the 1990s (6,7). Those studies showed favorable outcomes: (i) rates of cancer growth and new appearance of lymph node metastases (LNM) were low; (ii) no patients who underwent surgery form tumor growth or new LNM had life-threatening recurrence or died of thyroid cancer; and (iii) no patients developed distant metastases or died of thyroid cancer during AS (8–10).

Based on these findings, AS for low-risk PTMC was adopted as one of the management strategies in the 2010 guidelines for the clinical treatment of thyroid nodules by the Japan Association of Endocrine Surgeons and the Japanese Society of Thyroid Surgery (those two associations combined to form the present Japan Association of Endocrine Surgery [JAES] in 2018) (11). In 2015, this management strategy was consider as an alternative management strategy by the American Thyroid Association management guidelines for adult patients with thyroid nodules and differentiated thyroid cancer (12).

AS has been gaining a certain degree of consensus as an acceptable management option for low-risk PTMC worldwide. Indeed, according to a survey of the JAES member institutions in 2018, 53.8% of adult patients with low-risk PTMC at that time were undergoing AS in those Japanese institutions (13). However, many health care providers still have various concerns about providing AS in real-world practice. A task force on the management of PTMC created by the JAES thus produced the present consensus statements regarding indications and strategies for AS, to facilitate the implementation of this management alternative for adult patients diagnosed with low-risk PTMC.

## Methods

Members of the JAES task force on the management for PTMC were appointed by the board of the JAES and comprised 11 endocrine/thyroid surgeons, 1 head and neck surgeon, 1 endocrinologist, and 1 pathologist specializing in thyroid diseases. As the consensus method, we adopted modified nominal group process depending on the task force (14).

The relevant clinical questions were created with the input of task force members. The related literature in both English and Japanese languages through June 2019 was searched using search engines (PubMed, The Cochrane Library, and Ichushi web), and an additional hand search was also undertaken. We identified six clinical studies (8,15–19) regarding AS in adult patients with low-risk PTC (Table 1) but identified no randomized studies comparing outcomes between immediate surgery and AS. The statements were made based on scientific evidence as much as possible; and where evidence was lacking, principles considered appropriate, at least, at present were applied based on the consensus decision of members. Grading of evidence was not considered suitable for the present consensus statements.

**Table 1.** Clinical Studies of Active Surveillance in Patients with Low-Risk Papillary Thyroid Microcarcinoma

First Author, Country, Year	No. of patients	Duration of follow-up (months)	Tumor size enlargement (≥3 mm)	Tumor volume increase (>50%)	Development of lymph node metastasis
Ito, Japan, 2014 (8)	1235	Mean, 60	4.6% in total	ND	1.5% in total
			4.9%/5 years		1.7%/5 years
			8.0%/10 years		3.8%/10 years
Fukuoka, Japan, 2016 (15)	409	Mean, 81.6	6.0% in total	ND	1.0% in total
			6.3%/5 years		
			7.3%/10 years		
Tuttle, USA, 2017 (16)	291^a^	Median, 25	3.8% in total	12.4%	0%
			2.5%/2 years	11.5%/2 years	
			12.1%/10 years	24.8%/5 years	
Oh, Korea, 2018 (17)	370	Median, 32.5	3.5%	23.2%	1.4%
Sanabria, Colombia, 2018 (18)	57^a^	Median, 13.3	3.5%	ND	ND
Molinaro, Italy, 2020 (19)	93	Median, 19	2.1%	16%	1.1%

^a^ Included patients with papillary thyroid carcinoma 10–15 mm in diameter.

ND, no data.

## Diagnosis and Decision-Making

### Indications for AS and immediate surgery for PTMC

PTMC is defined as a PTC ≤10 mm in maximal diameter. Candidates for AS are adult patients with low-risk PTMC. Table 2 shows those PTMCs indicated for surgery. PTMCs with high-risk features including clinical LNM, distant metastasis (although very rare), and invasion to adjacent organs (particularly the trachea and recurrent laryngeal nerve [RLN]) should undergo immediate surgery, and postoperative radioactive iodine therapy should be given if indicated.

**Table 2.** Indication for Immediate Surgery in Papillary Thyroid Microcarcinoma Without Active Surveillance

1. Presence of clinical lymph node metastasis or distant metastasis (rare)
2. Clinically apparent invasion into the RLN or trachea
3. Diagnosis of aggressive subtype of papillary thyroid carcinoma on cytology (rare)
4. Tumors adherent to the trachea, possibly invading
5. Tumors located along the course of the RLN
6. Associated with other thyroid or parathyroid disease requiring surgery
7. Age <20 years (no current evidence)

RLN, recurrent laryngeal nerve.

Microscopic LNM have been detected at considerably high rates not only in the central neck compartment but also in the lateral neck compartments even in clinically node-negative PTMC, when these compartments were dissected prophylactically. However, based on the outcomes of patients who underwent AS, the rate of such microscopic metastases becoming clinically apparent appears low. Conversion surgeries performed following the appearance of clinical LNM were successful without compromising patient prognosis.

In addition, FNAC features suggestive of aggressive subtypes of PTC, such as tall cell variant, are recommended for immediate surgery, although direct evidence for this is lacking, and suspicion of such subtypes based on FNAC alone is not easy to diagnose in practice (20,21). Kuo *et al.* investigated surgical cases of 18,260 conventional PTMCs and found 97 tall cell variant of PTMCs, and 90 diffuse sclerosing variant of PTMCs (22). The investigators also found that diffuse sclerosing variant of PTMCs had extrathyroidal extension (ETE) and LNM significantly more frequently than conventional PTMC, and tall cell variant of PTMC had significantly larger tumors, and higher rates of tumor multiplicity and ETE than conventional PTMCs (22). Immediate surgery is also recommended for tumors adhering to the trachea or located along the course of the RLN, although these features are not necessarily biologically aggressive.

The revised JAES guidelines for the clinical practice of treating thyroid nodules published in 2018 recommended that AS should be performed by “an appropriate medical care team” (23). A multidisciplinary team including US specialists highly experienced in imaging of the neck region is mandatory for conducting AS to accurately evaluate primary tumors and lymph nodes at the beginning of and during AS, and to appropriately make a shared-decision with individual patients. Importantly, at present, evidence for the safety of AS in patients younger than 20 years is lacking.

**Key point:** Candidates for AS are adult patients with clinical T1aN0M0 low-risk PTMC.

### Evaluation of ETE and LNM

Tumors located on the ventral side of the thyroid and with US features suggestive of invasion into the strap muscles are not necessarily candidates for immediate surgery. Conversion surgery after progression of such cases would not impact the quality of life (QoL) of patients, because only partial resection of the muscles is required. Furthermore, such minimal ETE has very little influence on patient prognosis. PTMC located on the lateral capsule of the thyroid lobe rarely invade the carotid artery, allowing AS. However, for tumors on the dorsal side and suspected of invading into the trachea or RLN, the management strategy should be decided very carefully. To evaluate invasion into the trachea and RLN, not only US imaging but also computed tomography (CT) may be useful, especially in cases when tumor calcifications obscure the relationship between tumor and these structures. If needed, laryngofiberscopy is adopted to evaluate whether vocal cord paralysis is present (24–26).

**FIG. 1.**
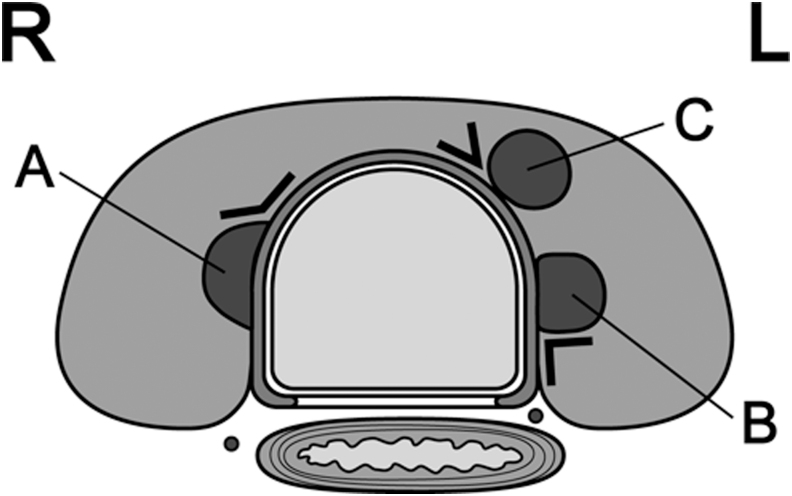
Angles between tumors and the trachea. **(A)** Obtuse angle; **(B)** almost right angle; **(C)** acute angle. L, left side; R, right side.

**FIG. 2.**
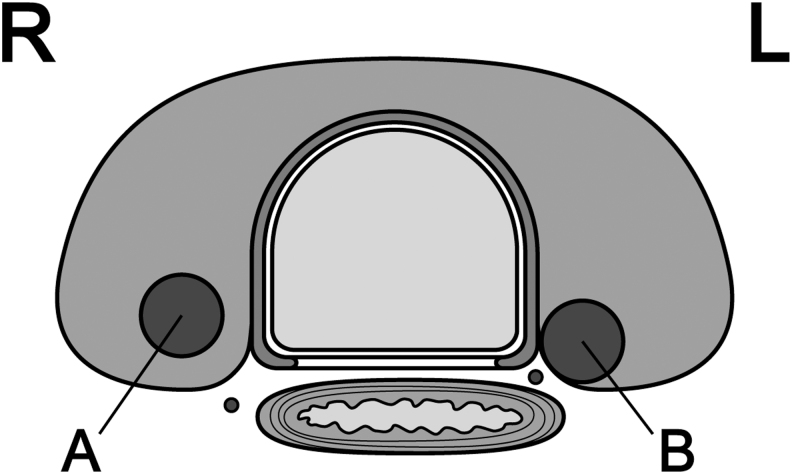
Presence **(A)** and absence **(B)** of a normal rim between the tumor and course of the RLN. The path of the RLN is more oblique on the right side than on the left side. Thus, tumors located away from the tracheoesophageal groove but in the dorsal region might still be at risk of invading the RLN. L, left side; R, right side; RLN, recurrent laryngeal nerve.

According to a report from Kuma Hospital (27), an obtuse angle between the tumor and trachea carries a high risk of invasion (Fig. 1). If there is no normal rim of thyroid tissue between the tumor and the course of the RLN, the RLN could be at risk of invasion (Fig. 2). No PTMCs <7 mm showed invasion into the trachea or RLN during surgery. However, 12 (24%) of 51 PTMCs ≥7 mm with an obtuse angle between the tumor and trachea required shaving of cartilage or resection of full layers of the trachea. Similarly, 9 (9%) of 98 PTMCs ≥7 mm without a normal rim between the tumor and the course of the RLN required shave resection, partial layer resection, or resection with reconstruction of the RLN during surgery. Tumors simply touching the trachea or far from the course of the RLN can be candidates for AS.

US is generally used for evaluating LNM. When LNM are suspected in the lateral compartment, FNAC with measurement of thyroglobulin from the needle washout is recommended. Lymph node swelling is frequently observed in cases with chronic thyroiditis, requiring careful consideration of the individual case (28). Ito *et al.* reported that PTMC located in the upper pole of the thyroid was more likely to show clinical or pathological lateral LNM (29), and Jeon *et al.* identified age <50 years, male sex, and microcalcification in the tumor as risk factors for lateral LNM (30). However, since there is no evidence that these risk factors other than young age are predictors of disease progression, cases showing these risk factors do not be consider as not candidates for AS.

**Key points:** ETE and LNM should be evaluated by US. CT imaging can be added if needed. For evaluation of invasion into the trachea and RLN, particular attention should be given to the angles formed between the tumor surface and trachea and the presence of a normal rim of thyroid tissue between the tumor and the course of the RLN.

### Evaluation of distant metastasis with chest CT

Regarding the incidence of distant metastasis from low-risk PTMC, Sugitani *et al.* reported that none of the 148 patients with T1aN0 PTMC showed distant metastasis or distant recurrence following surgical treatment (7). Ito *et al.* reported that distant metastases were not detected during follow-up in any of the 626 patients with T1aN0 who underwent surgery (6). Choi *et al.* reported that none of the 4927 low-risk PTMCs fulfilling the Memorial Sloan Kettering Cancer Center (MSKCC) indications for AS showed distant metastasis (31). Reinke *et al.* detected distant metastasis in 6 (0.7%) of 803 PTMCs, but none of the 527 T1aN0 PTMCs had distant metastasis (32). Furthermore, Kawano *et al.* reviewed 1000 consecutive patients with low-risk PTMC who underwent chest CT as a routine preoperative study. They reported that none of these patients had metastatic lesions on chest CT (33). Taken together, the incidence of distant metastasis in low-risk PTMC is extremely low, and routine chest CT may cause more harm than good.

As for the appearance of distant metastasis during AS, Sugitani *et al.* reported that none of their 230 patients showed distant metastasis (mean follow-up, 60 months) (9). Ito *et al.* likewise did not detect any distant metastasis in their 1235 cases of PTMC (mean follow-up, 60 months) (8). Tuttle *et al.* at the MSKCC detected distant metastasis in none of their 291 low-risk PTCs (defined as ≤1.5 cm) during AS (median follow-up, 25 months) (16), indicating that the incidence of distant metastasis appearing during AS for low-risk PTMC is also extremely low.

**Key point:** Chest CT is not indicated at initiation of AS but may be indicated if there is disease progression.

### Factors affecting decision-making in adult patients with PTMC

#### Age

A study from Kuma Hospital that enrolled 1235 cases of low-risk PTMCs under observation showed that the 10-year rate of progression (enlargement of tumor diameter to ≥12 mm or appearance of LNM) was 2.5% in patients aged ≥60 years, 4.9% in those aged 40–59 years, and 22.5% in those aged <40 years. Multivariate analysis revealed young age (≤40 years) as an independent risk factor for PTMC progression (odds ratio, 4.348; 95% confidence interval [CI 2.293–8.196]; *p* < 0.0001) (8). Miyauchi *et al.* estimated that the lifetime probability of disease progression was 48.6% for patients in their 20s at presentation, 25.3% in the 30s, 20.9% in the 40s, 10.3% in the 50s, 8.2% in the 60s, and 3.5% in the 70s (34). Other studies have also demonstrated a lower rate of PTMC progression in older patients (15–17,35).

**Key points:** Older patients with low-risk PTMC are ideal candidates for AS. Although tumors in young patients are more likely to progress, these patients can still be candidates for AS since the outcomes of surgery after AS are excellent and the lifetime probability of progression is <50% even among patients in their 20s at presentation.

#### Multiplicity

Since the initial AS report from Kuma Hospital in 2003, multiple lesions were not regarded as an exclusion criterion. Thirty (18.5%) of the 162 cases in AS had multiple lesions (6). A report by Ito *et al.* in 2010 found no significant difference in PTMC enlargement rates between multiple and solitary cases. Also, in 1055 patients who underwent immediate surgery, no significant association was observed between multiplicity and disease-free survival (DFS) rate (36). In 2014, they performed multivariate analysis of risk factors for PTMC progression during AS of 1235 cases, showing that multiplicity was not a risk factor for tumor enlargement by ≥3 mm or the appearance of LNM (8).

Sugitani *et al.* enrolled 571 low-risk PTMCs and performed AS for ≥1 year at the CIH and Nippon Medical School Hospital and found that 10-year progression rates of 115 multiple cases and 456 solitary cases were 14.8% and 12.2%, respectively (*p* = 0.51). Furthermore, multiplicity was not a risk factor for progression by multivariate analysis. In that study, 9 of the 10 patients with multiple lesions were obliged to undergo total thyroidectomy, and the authors concluded that AS has merit for multiple PTMCs to avoid total thyroidectomy and resulting surgical complications (37). Multiplicity is not listed as an exclusion criterion in protocols and reports on outcomes of AS for low-risk PTMC from the United States, Canada, or South Korea (28,38–40).

**Key points:** No data suggest that tumor multiplicity is associated with tumor enlargement and appearance of LNM. Patients with PTMC and multiple lesions can thus be candidates for AS.

#### Family history

Uchino *et al.* retrospectively analyzed 6458 surgical cases of differentiated thyroid carcinoma (DTC) and reported that 258 (4.0%) of these were familial DTC. These cases were more likely to be multiple and to show recurrence in the remnant thyroid, and DFS rates were lower than those of sporadic DTC. However, distant recurrence rates and overall survival rates did not differ between familial and sporadic DTC (41). Ito *et al.* reported that, of 6015 PTC surgical cases, 273 (4.5%) were familial PTC and clinicopathologic features (except for multiplicity), and DFS rates and cause-specific mortality rates did not differ between familial and sporadic PTCs. They concluded that the treatment strategy for familial patients should be the same as that for sporadic patients, except total thyroidectomy is recommended for familial patients (42).

Lupoli *et al.* analyzed seven cases of familial PTMC in 1999 and reported high rates of multiplicity and LNM (43). Capezzone *et al.* retrospectively analyzed 291 PTMC surgical cases (median follow-up, 8.3 years) and compared variables between 248 sporadic cases and 43 familial cases. Familial cases were found to be more likely to have pathological lesions in both lobes, to be positive for clinical LNM, and to have recurrence. They therefore concluded that family history should be given substantial consideration in the management of PTMC (44). However, Ito *et al.* found no significant difference in enlargement and appearance of LNM between familial and sporadic cases (8). The presence of a family history thus should not be a contraindication for AS. However, familial PTC may be more aggressive. When surgery is planned for familial cases after AS, total thyroidectomy should be considered.

**Key point:** PTMC in patients with a family history of PTC may be candidates for AS.

#### Desire to bear children and pregnancy

DTCs during pregnancy show favorable outcomes under appropriate management, and patients can safely deliver babies. Shindo *et al.* at Kuma Hospital analyzed low-risk PTMC patients during pregnancy and reported a higher rate of enlargement by ≥3 mm in pregnancy (4 of 9 patients, 44.4%) compared with that in an age-adjusted control group (3 of 27 patients, 11.1%; *p* = 0.0497) (45). However, the study cohort was small and the survey period was short.

Ito *et al.* at Kuma Hospital subsequently investigated whether female patients with low-risk PTMC capable of pregnancy can be candidates for AS by increasing the number of cases with a longer study period. In this report, 50 patients with low-risk PTMC had 51 pregnancies during AS. Although 4 patients (8%) showed an increase of ≥3 mm in diameter of the PTMC, no change in size was observed in the remaining 46 (92%). Of the four enlarged cases, two underwent surgery after delivery and had no recurrence. The remaining two underwent continuous AS because of the lack of enlargement after delivery (46).

**Key point:** Since careful and constant surveillance enables safe management of low-risk PTMC in pregnancy, patients desiring to bear children and pregnant patients can be candidates for AS.

#### Calcification

Fukuoka *et al.* reported that the degree of calcification was associated with the enlargement of PTMCs. They investigated this issue in 384 patients (484 lesions) who underwent AS for a mean of 6.8 years, by dividing patients into four categories of calcification: none, micro, macro, and rim (15). The mean age in each category was 52.1, 54.2, 56.3, and 60.1 years, and the rate of enlargement by ≥3 mm was 9.6%, 5.5%, 3.2%, and 0%, respectively. Degree of calcification correlated significantly with patient age, and tumors with coarse calcification were less likely to enlarge. Also, during AS, 25.1% and 51.8% of lesions showed an increase in the degree of calcification at 5- and 10-year follow-up, respectively. On multivariate analysis, cases with coarse calcification (macro or rim) in the most recent examination showed a significantly lower enlargement rate (odds ratio, 0.34 [CI 0.11–0.87]; *p* = 0.022).

In a study from Kuma Hospital (47), 180 patients who underwent surgery after one-year of AS, 13.8% of 160 nonenlarged cases during AS showed macrocalcification ≥2 mm with acoustic shadow, while none of the 18 enlarged cases displayed this. In contrast, Oh *et al.* from South Korea studied 273 low-risk PTMCs observed for ≥1 year (median, 42 months) and demonstrated that cases with macrocalcification at initiation of AS were more likely to show an enlargement of tumor volume by ≥50% compared with those with microcalcification, discordant to reports from Japan (35). Taken together, low-risk PTMCs with weak calcification could progress compared with nodules with strong calcification, but calcification is reported to become strong with time. PTMCs with fine or no calcification are thus still candidates for AS.

**Key point:** There is no convincing evidence to exclude patient with PTMCs based on the degree or type of calcification from considering AS as a management strategy.

#### Vascularity

In 2010, Sugitani *et al.* (9) at the CIH demonstrated that in AS of 230 patients (300 lesions) for an average of five years, tumors with rich vascularity (33 lesions, 12%) showed a higher rate of enlargement by ≥3 mm than those with poor vascularity (245 lesions, 88%) (30.3% vs. 3.7%; *p* < 0.0001). Furthermore, in 2016, Fukuoka *et al.* at the same institution investigated the relationship between change in vascularity over time in each tumor and prognosis (15). According to their data, 14.6% of 70 lesions with rich vascularity at initiation of AS enlarged by ≥3 mm, significantly higher than the rate of enlargement in 410 lesions with poor vascularity (4.6%). Also, vascularity decreased during AS in 61.4% of PTMCs with rich vascularity at initial evaluation. On multivariate analysis, cases with poor vascularization at the most recent examination showed significantly lower tumor enlargement rates (odds ratio, 0.17 [CI 0.07–0.43]; *p* = 0.0004). Therefore, although tumors with rich vascularity have a higher risk of enlarging than those with poor vascularity, vascularity in low-risk PTMCs decreases over time.

**Key point:** Evidence is lacking for excluding PTMCs based on the degree of vascularity from considering AS as a management strategy.

#### Coexistence of Graves' disease and benign nodules

No reports appear to have investigated the influence of Graves' disease on PTMCs under AS. Conclusions are discrepant regarding the prognosis after surgery for PTC with Graves' disease (48–51). Whether the coexistence of Graves' disease affects the biological behavior of PTMCs remains unclear. However, at least there is no evidence that such cases should be excluded as candidates for AS. Changes in thyrotropin (TSH) levels due to the treatment for Graves' disease may influence tumor enlargement during AS.

Regarding coexisting benign nodules, although surgery might be performed in the future due to the enlargement of benign nodules, no evidence is available for excluding AS for PTMCs in patients with benign nodules.

**Key points:** No evidence exists that patients with Graves' disease or benign nodules should be excluded for AS of PTMC. Management strategies should be decided according to the surgical indications of the coexisting diseases.

## Follow-Up and Monitoring

As shown in Table 1, a small number of PTMCs under AS show progression, namely tumor enlargement or new LNM appearance. Follow-up examinations are thus mandatory to avoid overlooking the need for surgery due to disease progression. Table 3 summarizes indications for surgery during AS.

**Table 3.** Indications for Surgery After Active Surveillance for Papillary Thyroid Microcarcinoma

1. Tumor diameter reaches 13 mm
2. Appearance of new lymph node metastasis
3. Change in patient preference
4. Appearance of other thyroid disease or parathyroid disease requiring surgery

### Appropriate interval between examinations

In the protocols of prospective studies of AS performed by two Japanese institutions, US examination was performed twice a year at initiation of AS, followed by at least once a year thereafter. Experienced examiners evaluated changes in tumor size and appearance of new thyroid lesions and LNM (8,15). Other protocols for AS recommend US examination once or twice a year (39), or examination every six months within two years after initiation of AS, and thereafter, if nothing has changed, once every one or two years (28,38,40). Although no comparative studies have been published regarding the optimal interval for US examination, most prospective studies have adopted examination once every six months or one year for follow-up. No evidence is available regarding when the interval between examinations can be further extended, such as to every 2–3 years.

**Key point:** US evaluations of PTMCs by experienced examiners are recommended every 6 months for 1–2 years after initiation of AS and once a year thereafter if no disease progression is detected.

### Definition of tumor enlargement and proper timing for surgery

Tumor size is measured by US on sagittal and coronal section images, and the largest value is generally regarded as the maximal diameter. Enlargement was defined as an “increase in maximal diameter by ≥3 mm” by Ito *et al.* in 2007 (52). In 2010, Ito *et al.* (36) and Sugitani *et al.* (9) reported the rate of enlargement under this definition for long-term observation. This definition is simple and thus has been adopted by many centers. Using this definition, no patients showed RLN paralysis or distant metastasis during AS.

Recently, some researchers have defined enlargement as a 50% increase in tumor volume by measuring three dimensions (16,17,39). Another report found that the doubling time for tumor volume offers a highly useful marker of tumor growth rate (35). They reported that evaluation of tumor volume can accurately reflect changes in tumor size and can be used as a potential prognostic marker of future growth as used in other malignancies. However, this approach appears overly sensitive, as the growth rate of a nodule growing from 6 × 6 × 6 to 7 × 7 × 7 mm is 59%. Neither of the two Japanese institutions, which regard an increase in maximal diameter ≥3 mm as enlargement, has reported cases in which surgical intervention was too late. Measurement of tumor volume is subjected to a high degree of observer variability since US is a bidimensional technique and the volume is extrapolated with all the potential pitfalls of such a measure (53). As a result, evaluation based on maximal diameter is recommended as a practical and simple method.

As for the timing of surgery, Miyauchi and Ito noted that AS could be allowed to continue until tumor diameter reached 13 mm if the patient preferred (24), since a 3-mm increase in the largest PTMC (i.e., 10 mm in maximum diameter) makes a 13-mm tumor. Sakai *et al.* reported that 5- and 10-year disease progression rates of T1bN0M0 PTC (maximal diameter, 11–16 mm; mean, 11.7 mm) were 5% and 12%, respectively, showing no difference from those of T1aN0M0, and none of the PTC T1bN0M0 < 15 mm showed recurrence after surgery (54).

Miyauchi *et al.* performed a kinetic analysis of tumor volume from 169 PTMCs under AS and reported that only 3% showed rather rapid growth (doubling rate >0.5/year), 17% even shrank (<0.1/year), and the incidence of enlarged nodules decreased with age (55). Regarding the clinical course of PTMC after enlargement, Ito *et al.* analyzed a series of 824 low-risk PTMCs and reported that, of tumors that once showed an enlargement in maximal diameter ≥3 mm and in tumor volume ≥50%, only 7.7% and 3.8% showed further enlargement, respectively. Growth after enlargement decreased significantly compared with before enlargement, and many cases showed tumor shrinkage (56). These findings suggest that surgery for PTMCs immediately after the diameter exceeds 10 mm is not always necessary. For such cases, continuous AS can be performed in consideration of tumor location (possibility of invasion of the RLN and trachea), velocity of growth, and patient preference.

**Key points:** To evaluate tumor growth, maximal tumor diameter should be used and an increase of ≥3 mm on US is considered to represent enlargement. Immediate surgery when maximal PTMC diameter exceeds 10 mm is not always necessary.

### Should TSH suppression therapy be performed during AS?

No randomized prospective trials have been published about TSH suppression therapy during AS for low-risk PTMC, but some reports have been published regarding the relationship between TSH levels and tumor enlargement. Sugitani *et al.* (57) reported that TSH levels at diagnosis and average TSH levels during AS (mean follow-up, 6.5 years) of 323 patients (415 lesions) did not differ between the enlargement group (≥3 mm) and the nonenlargement group. On multivariate analysis, TSH at diagnosis was not regarded as a significant predictor of tumor enlargement, and the average TSH level during AS was not significantly associated with change in tumor volume.

In contrast, Ito *et al.* reported (8) that, in a series of 1235 cases (mean follow-up, 60 months), 57 (4.8%) of 1184 patients who did not undergo TSH suppression therapy showed tumor enlargement, while PTMC in only 1 (2.0%) of 51 patients who underwent TSH suppression enlarged. Although the difference was not significant, none of the patients on TSH suppression therapy showed progression to clinical disease (the tumor size reaching 12 mm or larger or the new appearance of LNM), and they suggest TSH suppression therapy as possibly preventing low-risk PTMC progression. Kim *et al.* in South Korea (58) reported that, in AS for 126 patients (median follow-up, 26 months), the high TSH group had a higher rate of tumor volume increasing by ≥50% than the other groups. They reported high TSH level as an independent risk factor for PTMC enlargement, and progression-free survival rates were significantly worse for low-risk PTMC with TSH >2.50 mIU/L than for other groups. Based on the above, the possibility of high TSH level affecting low-risk PTMC progression cannot be ruled out, but at least at present, it is not possible to conclude that TSH suppression therapy is beneficial for patients. Active TSH suppression might have adverse effects on the heart or bone, particularly for older patients (59). Keeping the TSH level at a low normal level to avoid excess stimulation could be acceptable for young patients, in whom PTMCs are more likely to enlarge.

**Key points:** Insufficient evidence is available regarding the effect of TSH suppression therapy during AS for low-risk PTMC. When performed, keeping TSH levels at low normal may be best.

### How long and how many years should AS be continued?

As described before, the progression rate of low-risk PTMC is considered to decrease with age. However, older age is one of the most important factors predicting poor prognosis (60). With a low probability, if PTMC in an older patient progresses and is not detected early, a life-threatening situation may develop. At present, no evidence is available regarding when and at what age AS can be stopped.

Continuation of AS throughout life does not represent a disadvantage of AS, because even after surgery, in addition to regular postoperative follow-up, administration of thyroid hormone could be needed. Oda *et al.* compared 10-year medical costs of management between immediate surgery and AS. Costs were reportedly about 4.1 times higher in the immediate surgery group than in the AS group in the Japanese medical system (61). In a report from Hong Kong, medical costs of the AS group were lower for 16 years after initiation of treatment and cost performance was even better thereafter compared with the immediate surgery group (62).

**Key points:** The incidence of low-risk PTMC progression could become lower with age. However, if PTMCs in older patients progresses, they might have a poor prognosis. There is no evidence regarding after how many years AS can be discontinued. AS throughout life is therefore recommended.

## Topics for Future Research

### Molecular markers

Common genetic alterations in PTC include *BRAF*, *RAS*, and *RET/PTC* mutations. *BRAF* gene mutation is the most frequent one, and some investigators have reported that this mutation in PTMC is associated with ETE, LNM, and recurrence (63–66). The combination of *BRAF* and *TERT* promoter mutations was associated with poor prognosis in clinical PTCs (67). Yabuta *et al.* examined surgical specimens of PTMC removed after AS and reported that the rate of *BRAF* gene mutations did not differ significantly by stable disease group (61%), tumor enlargement group (70%), and LNM appearance group (80%). None of these cases were positive for *TERT* promoter gene mutations (68). While de Biase *et al.* reported that 4.7% of 431 PTMCs had *TERT* gene mutations. However, these mutations were not associated with aggressive features or clinical outcome in their cohort (69).

**Key point:** Currently, reliable molecular markers of PTMCs behavior are lacking.

### Patient-reported outcomes

The perspective of patients represents important information. Choosing AS can avoid complications and adverse events of surgery, but anxiety regarding disease progression may arise in patients. In contrast, surgery might decrease anxiety about “cancer” because the patient is “cured,” but side effects of surgery may persist.

Regarding anxiety in patients with thyroid cancer, Hedman *et al.* found that 48% of patients diagnosed with DTC reported anxiety regarding recurrence, even though 14–17 years had already passed (70). Sawka *et al.* reported that, even for PTMC patients, anxiety regarding the disease affects their daily lives to some extent (71–73). Smulever *et al.* showed that only 26 (19%) of 135 patients with PTC ≤15 mm chose AS at the time of cytological diagnosis, with anxiety given by many patients as a reason for selecting surgery (74). In contrast, Davies *et al.* performed a mixed-method survey study with semi-structured interviews and field observation in 234 patients who underwent AS at Kuma Hospital. Although 37% of patients felt uneasy regarding cancer progression, 60% answered that their anxiety had decreased compared with the early days of AS. In addition, 83% of patients reported AS as the best management strategy for them personally (75).

A limited number of studies have been published comparing anxiety between patients who underwent AS and those who received immediate surgery. Yoshida *et al.* compared anxiety between 20 patients who underwent AS and 30 patients who had surgery in a cross-sectional study using the State-Trait Anxiety Inventory and found that patients who chose AS tended to have a deeper anxiety than those who had surgery at the time of the survey. However, the degree of anxiety correlated with each patient's trait anxiety, not with the choice of management (AS or immediate surgery) and correlated inversely with the period of time (76).

Jeon *et al.* compared QoL between 43 patients who underwent AS and 148 patients who underwent lobectomy using the 12-item short-form (SF-12) questionnaire in a cross-sectional study and reported that the psychological burden in the AS group was less than that in the lobectomy group (coefficient, −7.71 [CI −15.26 to −0.16]; *p* = 0.045) (77). Kong *et al.* longitudinally compared 203 patients who underwent AS with 192 patients who surgical treatment using a QoL questionnaire specific for thyroid. They reported that the AS group was superior to the surgical group with respect to psychological health at the beginning of investigation and in psychological and physical health at the end of the eight-month investigation (78).

AS appears superior to surgery in terms of physical QoL, but anxiety could be stronger than that was observed with immediate surgery. With appropriate explanation and the attitude of health care providers, patient anxiety is considered to gradually decrease, but long-term comparative studies regarding patient-reported outcomes (PROs) are required for both AS and surgery in the future. Considering the values and preferences of patients and providing appropriate information with an appropriate attitude is crucial in the shared decision-making and follow-up process (79).

**Key point:** Evidence for PROs regarding the management of low-risk PTMC is still insufficient, requiring long-term comparative studies in the future.
